# An evaluation study of caregiver perceptions of the Ontario’s Health Links program

**DOI:** 10.1371/journal.pone.0229579

**Published:** 2020-02-27

**Authors:** Ruta K. Valaitis, Maureen Markle-Reid, Jenny Ploeg, Michelle L. Butt, Rebecca Ganann, Nancy Murray, Sue Bookey-Bassett, Laurie Kennedy, Claudia Yousif

**Affiliations:** Aging Community and Health Research Unit, School of Nursing, Faculty of Health Sciences, McMaster University, Hamilton, Ontario, Canada; Columbia University, UNITED STATES

## Abstract

**Introduction:**

In 2012, the Ontario government launched Health Links (HL), which was designed to integrate care for patients with multimorbidity and complex needs who are high users of health services. This study evaluated perceptions of family and friend caregivers of patients enrolled in the HL program. Research questions included: What are (a) characteristics of caregivers of patients enrolled in HL (b) caregivers’ perceptions of the program in relation to HL’s guiding principles (patient and family-centred care, accessibility, coordination of services, and continuity of care and care provider) and (c) caregivers’ perceptions of the impact of HL on themselves and their care recipient?

**Methods:**

This study involved a survey and qualitative, semi-structured interviews. HL guiding principles (patient and family-centered care, accessibility, coordination of services, and continuity) guided the analysis.

**Results:**

Twenty-seven surveys and 16 qualitative interviews were completed. Caregivers reported high levels of strain [Modified Caregiver Strain Index (MCSI) 15.5 (SD 7.03)], mild anxiety [Generalized Anxiety Disorder (GAD 7), 9.6 (SD 6.64)] and depression [Center for Epidemiological Studies Depression Scale (CES-D 10), 11.9 (SD 8.72)]. Regarding the guiding principles, most caregivers had a copy of the HL patient’s care plan, although some caregivers noted that their needs were not included in the plan, nor were they asked for input. Caregivers found the program’s home and phone visits accessible. Despite minimum wait times for community-based services, other access barriers persisted, (i.e., out-of-pocket costs). HL provided well-coordinated patient services, although some perceived that there was poor team communication. Caregiver perceptions varied on the quality of care provided. Provider continuity provided caregiver relief and patient support: A lack of continuity was related to changes in care coordinators and weekend staff and attrition.

**Conclusions:**

Caregivers of HL patients appreciated patient- and family-centred, accessible, consistent, coordinated and team-based approaches in care. Providers and decision-makers are urged to ensure that programs aimed at high system users address these core concepts while addressing caregivers’ needs.

## Introduction

Living with multiple chronic conditions (MCC) has been associated with high use of health care services, including physician visits, emergency department visits and hospitalizations.[1, 2] In Canada, much attention is given to the high users of the health care system who make up 5% of the population and account for up to two-thirds of healthcare use and costs.[3, 4] It has been argued that integrated care models should be targeted to patients with patterns of health care use that could most benefit from coordination.[3] Modeled after accountable care organizations in the US, England, Australia and New Zealand, the province of Ontario’s Ministry of Health and Long-Term Care launched the Health Links (HL) program in 2012. HL aims to integrate care for all high users of hospital and emergency services who have multiple chronic conditions (≥4) and complex needs.[5]

There are 82 HL programs across 14 regional health authorities [i.e., Local Health Integration Networks (LHINs)] in the province of Ontario.[6] The role of the HL programs is to implement the Ministry’s goal of improving seamless and ongoing care coordination with a coordinated care plan, connecting patients to primary care providers, and engaging patients in their own health care using a patient and family-centred approach.[6] The HL program involves each patient having a professional care provider who creates an individualized and coordinated care plan for the HL patient and is their key point of contact to manage their care. The expectation is that the care provider will be familiar with the patient’s plan, medical history and ensure that their plan is being followed.[7]

The Ministry of Health and Long-term Care commissioned this study to evaluate caregivers’ perceptions of the HL program, although no HL program goals or objectives are explicitly directed at caregivers. The Ministry in no way influenced the results or the reporting of the study results. Despite the perceived rewards from caregiving such as personal growth and fulfilling commitment to a loved one [8], caregivers also experience multiple health concerns, such as depression, anxiety, social isolation and fatigue.[8–10] Satisfaction of caregivers of frail older adults has been associated with having resources to help care for their loved one, information and advice on illness, in-home respite, and counselling.[11] However. financial barriers and an inability to navigate the healthcare system have hindered caregivers from accessing formal services.[12] Caregivers face increased complexity when managing declining health status and well-being in their loved one and often face gaps between needs and health and social service supports to meet these needs [13]. Designing care processes that integrate patients and caregivers as care recipient dyads is required to better address gaps in needs and services for high system users and their caregivers. [8, 14] To best support caregivers who care for frequent users of the healthcare system, we must understand their perspectives.

Family and friend caregivers (hereafter referred to as caregivers) are an integral part of keeping patients stable in their homes.[15] Although researchers have explored caregiver needs quite extensively, perceptions of caregivers of patients who are frequent users of the healthcare system have not been well examined. The purpose of this study was to evaluate perceptions of caregivers’ of HL patients of the HL program. Results can inform health and social service providers, decision-makers, and policy makers about how programs such as HL can support caregivers of patients who have complex needs. The following research questions were explored: (a) What are characteristics of caregivers of patients enrolled in HL and the care they provide? (b) What are caregivers’ perceptions of the program in relation to HL program’s guiding principles (patient- and family-centred care, accessibility, continuity of care and care provider, and the coordination of services), and (c) What are caregivers’ perceptions of the impact of HL on themselves in their caregiving role and the HL patients to whom they provide care?

## Methods

### Design

Full ethics approval was obtained from the Hamilton Integrated Research Ethics Board (HiREB) for Hamilton Health Sciences and McMaster University’s Faculty of Health Sciences [HIREB #2104]. A survey was conducted first to determine the length of time caregiving, type of care received, satisfaction with and impact of care received, and caregivers’ health status in terms of caregiver strain, anxiety, depressive symptoms, social support and demographic characteristics of caregivers of HL patients (e.g., age, gender). This was followed by qualitative semi-structured individual interviews with caregivers of HL patients to understand their perceptions of the program including perceived benefits, needs, and impacts of HL on themselves and the HL patients for whom they care.

**Fig 1:**
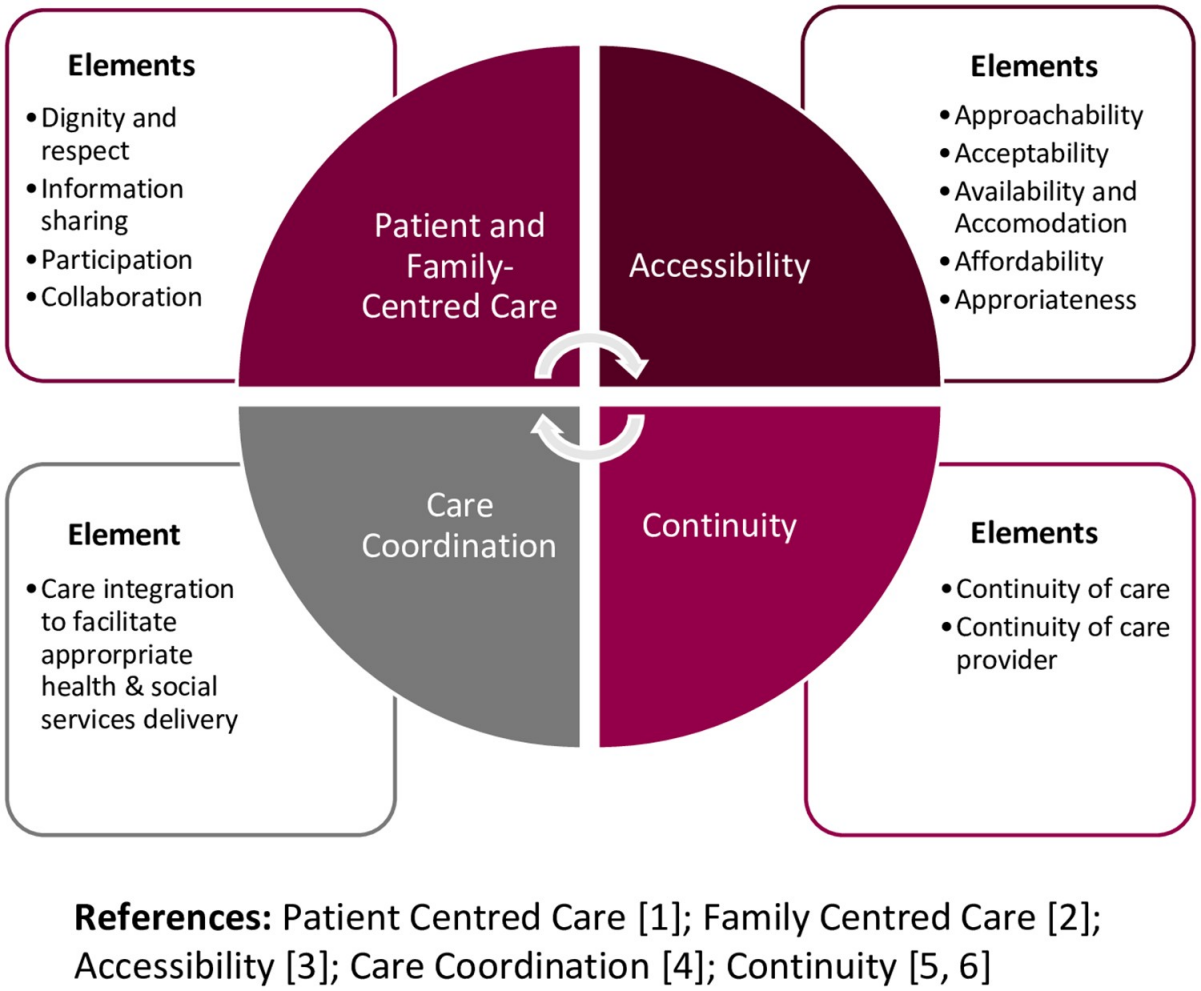
Health links core concepts.

Four core concepts, derived from the guiding principles for HLs [7] were used to inform data collection and analysis: 1) patient and family-centred care, 2) accessibility, 3) continuity of care and care provider, and 4) coordination of services (Fig 1). Definitions of each concept and its elements are supported by literature and can be found in S1 Table.

### Sampling and recruitment

Our research team worked in collaboration with researchers at Queen’s and Western Universities who were concurrently conducting a study exploring the HL providers’ and patients’ perceptions of the program, respectively. Using the Ministry of Health and Long-term Care’s facilitated leadership approach, each region adapted the implementation of HL program in their community to meet their unique needs with available resources.[5] Our teams agreed on a maximum variation sampling strategy that targeted diverse populations living in different regional health authorities (LHINs) in Ontario in order to capture a range of perceptions from services offered by different LHIN providers. These regions represent various geographic, rural and urban, socio-economic, and ethno-cultural communities which can take into account the influence of intersectionality.[16] The partner university team identified patient participants from three different regional health authorities, which included six HLs programs: 1) the South East LHIN (including HL programs in Quinte, Thousand Islands, and Rural Hastings); 2) the Central LHIN (including HL programs in South Simcoe and Northern York Region, and North York Central); and 3) the Hamilton Niagara Haldimand Brant LHIN (including the HL in Niagara North East).

HL patients in the patient perspectives study led by Queen’s University were asked to share the names of their primary caregiver. These caregivers were then contacted by phone to participate in this study. Caregivers were eligible if they were English-speaking, mentally competent, and were an adult caregiver of HL patients. A 10-item pretest questionnaire was conducted to determine mental capacity of caregivers to consent to participate using the Short Portable Mental Status Questionnaire (SPMSQ).[17, 18] Participants were required to have 5 or more correct answers of the 10-item questionnaire to participate (5–7 errors = moderate cognitive impairment). Verbal consent was obtained by the research coordinator by phone.

Participants were informed of the study purpose. The researchers had no relationships with Health Links participants prior to study commencement nor did they have any affiliation with the delivery of the Health Links program. A total of 27 caregivers from 6 HL programs in 3 LHIN jurisdictions completed the quantitative survey. At the end of the quantitative survey, caregivers were invited to participate in a qualitative telephone interview. For the qualitative component, purposive sampling was used to obtain a sub-set of participants with a target sample size of 15 to 20 caregivers. A sampling matrix was used to obtain a diverse sample of caregivers from the participating HL sites and a mix of male and female caregivers who were ≥ 65 and ≤ 65 years of age. All caregivers who were invited to participate agreed to be interviewed for the qualitative component (n = 16). No interviews were repeated to obtain clarifications.

### Data collection

Data collection was conducted between November 2016 and March 2017. A survey was developed to obtain descriptive characteristics of caregivers of HL patients and HL patients and caregiver perceptions of the program. The 70-item quantitative survey was comprised of: a) participant demographic characteristics (e.g., age, gender, marital status, education); b) caregiver roles (e.g., length of time caregiving), and formal and informal supports received; c) the HL patient’s demographics and health problems; d) four reliable and valid instruments to assess caregivers’ strain, anxiety, depressive symptoms, and social support; e) questions developed by this research team related to four core concepts: patient and family-centred care, accessibility, continuity of care and care provider, and coordination of services, as well as perceived impact of HL on caregiver health and well-being. (See S1 File).

The four validated instruments included: the Modified Caregiver Strain Index (MCSI) [19], the Generalized Anxiety Disorder (GAD-7) Scale [20], the Center for Epidemiological Studies of Depression–Short Form (CES-D 10) [21], and the abbreviated, 11-item Duke Social Support Index (DSSI).[22] The MCSI has strong reliability (internal consistency reliability: alpha = .90, and a two-week test-retest reliability coefficient of .88.[19] Validity has been examined with appropriate correlations reported between the MCSI and other instruments or variables associated with caregiver stress and strain.[19, 23] The GAD-7 is a reliable and valid measure of anxiety in the general population.[24] The CES-D 10 has high internal consistency (alpha = .78) [25] and test-retest reliability [26]; as well as acceptable validity testing results.[21] The reliability and validity of the DSSI has been established in a sample of community dwelling older adults.[27]

The research team also developed survey questions including structured (Likert-style) and open-ended questions that explored caregivers’ perceptions of the impact of HL on themselves and their care recipients in relation to the four guiding principles of the HL program to support the evaluation, as well as caregivers’ health and quality of life. Perceived impacts were measured using 15 statements on a 5-point scale (1 [*strongly disagree*] to 5 [*strongly agree*]) (see S2 Table: Caregiver’s Perceived Impacts of HL on Themselves and the HL Patients for Whom They Provide Care (n = 27)).

Statements targeted caregivers’ perceptions in relation to HL assistance in helping to care for their loved one, and HL program assistance to them as a caregiver. Additionally, overall satisfaction was measured with two statements using a 5 point scale ([1- very dissatisfied to 5- very satisfied]): “Overall, how satisfied are you with HL as a caregiver?’ and, “Overall, how satisfied are you with HL for your loved one/friend.” The full questionnaire was pretested with two older adult HL caregivers to determine clarity, comprehensibility, and to gauge the expected time for completion. No changes were made based on feasibility testing results. The quantitative survey took about 30 to 45 minutes to complete.

After participants consented to an qualitative interview, audio-recorded telephone interviews were conducted at another time by two female PhD-prepared registered nurses who have conducted multiple qualitative research studies (NM- the Project Coordinator; and RV—Professor) (see S2 File: Health Links Caregiver Evaluation Study Interview Guide). Field notes were captured to obtain interviewers’ reflections. Qualitative interviews lasted 60 to 90 minutes. Participants were given a $20 gift card for survey participation and a $50 gift card for completing the qualitative interview. Member checks were conducted by telephone with 2 caregivers to validate our interpretation of the qualitative data.

### Data analysis

Quantitative data analyses were performed using the statistical software IBM^®^ SPSS^®^.[28] Surveys were completed and anonymized responses were entered into a secure online database (Limesurvey). Responses were summarized using descriptive statistics; frequencies for nominal or ordinal data; and means with standard deviations as measures of central tendency and dispersion, respectively, for interval/ratio (continuous) data. Responses to individual questionnaire items specifically targeting caregivers’ perceptions of HL reported on a 5-point Likert scale (*1* [*strongly disagree*] to *5*[*strongly agree*]) were treated as continuous data. Total scale scores (or subscale scores, where relevant) were utilized to summarize caregivers’ responses on psychometrically tested instruments used within the HL Evaluation: Caregiver Survey.

Using a qualitative descriptive approach [29, 30], an inductive and deductive analysis approach was used to develop an understanding of HL caregivers’ perceptions of the program, perceived benefits, gaps and impacts of this service delivery model. Line-by-line inductive coding was conducted using N-Vivo 10 software [31] to identify first level descriptive codes [32]; these codes were then deductively organized under HL core concepts that were informed by HL guiding principles (Fig 1). The first level codes were then collapsed into pattern themes under each core concept.[32] Positive and negative perceptions were grouped under each theme to identify where there were differences and similarities among caregivers. Analysis was conducted concurrently with data collection to allow for further exploration of emerging themes in subsequent interviews. The first three interview transcripts were initially coded by NM and reviewed by RV with adjustments made through consensus. This resulted in a preliminary coding structure.[33] The interviews and coding structure were then reviewed by the full research team at a face-to-face meeting to reach agreement on the approach. The remaining coding was conducted by NM and again reviewed by RV (by checking quotes against codes using coding stripes in NVivo). The final coding structure was shared with the research team over multiple meetings to reach agreement on the themes. Although caregivers described varied perceptions of the quality of the program, data saturation was obtained as no new ideas were raised by caregivers to inform new themes.

## Results

Quantitative and qualitative results were integrated into the following sections: (a) characteristics of HL caregivers and caregiving (b) characteristics of HL patients, (c) HL core concepts, (d) perceived impacts of HL on patients and caregivers and, (e) recommendations for HL proposed by caregivers. The [ID #] of participants is noted where quotes are used to support results.

### Characteristics of caregivers

**Fig 2:**
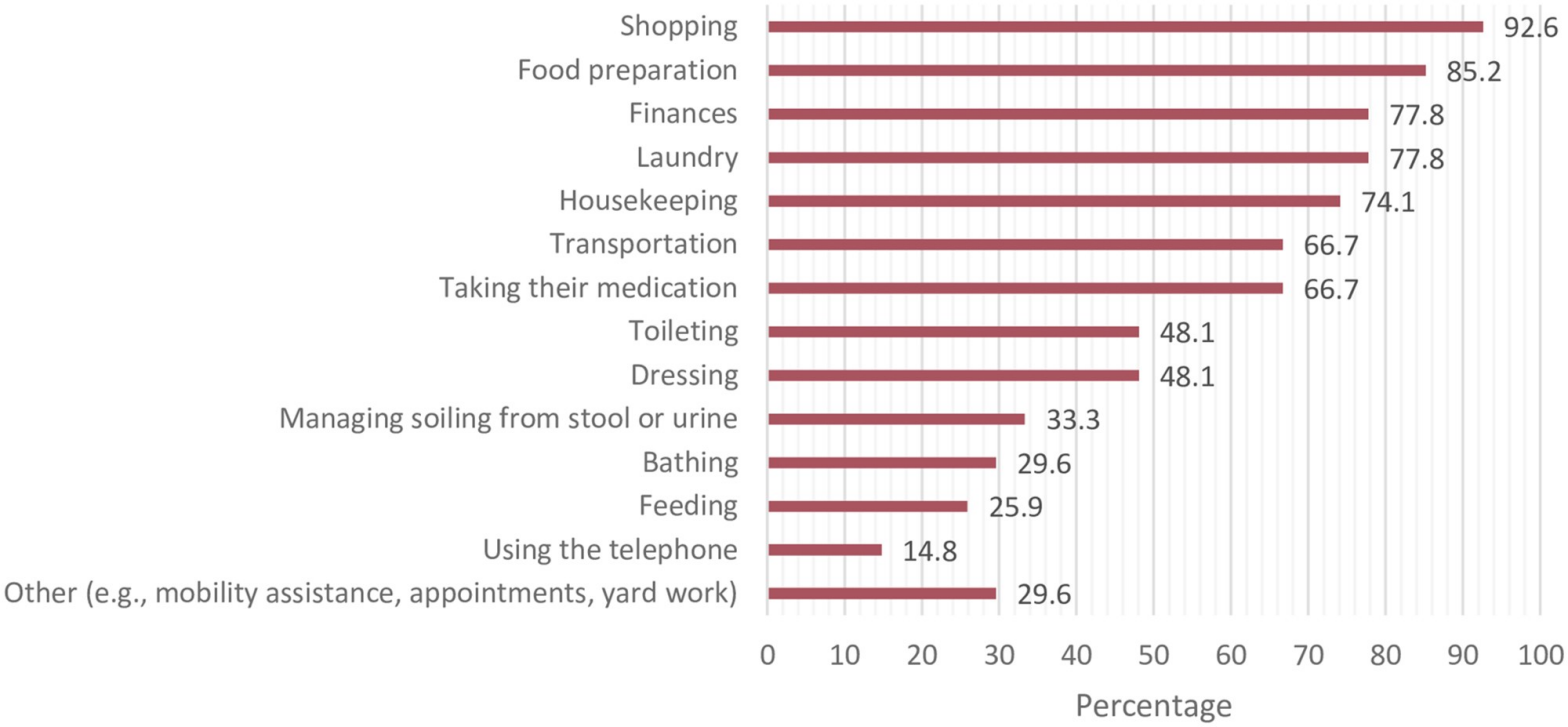
Types of assistance provided by caregivers (n = 27).

Table 1 presents demographic characteristics of participants and characteristics of their caregiving (n = 27). Most of the caregivers were the spouse/partner of the HL patient (51.9%) or the patient’s son or daughter (37.0%) and provided care on a “live-in” basis (74.1%). They spent a mean of 4.8 years (range 0.2 to 50 years) providing care to the HL patient. Fig 2 illustrates types of assistance that caregivers provided based on survey items. Most frequently reported caregiving activities were instrumental activities of daily living (IADLs), such as shopping and food preparation, which was supported by qualitative data. A caregiver noted that “*duties include 85% of the cooking and all the housework plus helping with care*” [ID 13]. With respect to activities of daily living (ADLs), caregivers most frequently assisted with toileting (48.1%) and dressing (48.1%). Open-ended survey responses indicated that most caregivers provided general support for their loved one, coordinated their care, and assisted with tasks around the home. One caregiver expressed the effort required: *“…only through me being diligent and being on the phone and asking questions and finding out what was available… did I attain all the information [related to supports] for my loved one*” [ID 8]. Some participants identified advocacy as another significant caregiving role. IADLs and ADLs were identified as part of the caregiver role in the survey as well as qualitative results.

**Table 1:** Demographic characteristics of participants (n = 27).

Characteristics of Participants			
	Mean	SD	Range
Age in Years	65.5	9,26	51–85
No. of Months Care provided to the HL Patient	58	110.7	2–600
**Gender**		**n**	**%**
Male		12	44.4
Female		15	55.6
**Marital Status**		**n**	**%**
Married		19	70.4
Single		5	18.5
Divorced		1	3.7
Other		2	7.4
**Highest Level of Education**		**n**	**%**
Elementary School		2	7.4
Some High School		3	11.1
Completed High School		8	29.6
Trades Diploma		2	7.4
College Diploma		7	25.9
Undergraduate Degree		5	18.5
Master’s Degree		0	0
Doctoral Degree		0	0
**Work**		**n**	**%**
Worked Outside the Home		9	33.3
Providing Care and/or Support to Dependents		5	18.5
**Social Assistance**		**n**	**%**
Receiving Social Services		4	14.8
Receiving Old Age Security caregivers ≥ 65 years of age		11	84.6
**Health Link Site**		**n**	**%**
Quinte Health Link		7	25.9
Thousand Islands		4	14.8
Rural Hastings		4	14.8
South Simcoe & Northern York		4	14.8
North York Central		6	22.2
Niagara North East		2	7.4
**Relationship to HL Patient**		**n**	**%**
Spouse		14	51.9
Son/Daughter		10	37.0
Niece/Nephew		1	3.7
Other		2	7.4
**Lives with the HL patient**		**n**	**%**
Yes		20	74.1
No		7	25.9

Approximately half (51.8%) of the participants used personal funds to support their caregiving activities. Survey results indicated that 85.2% of caregivers received some formal assistance in their caregiving activities (e.g., paid service providers, a support program, or a group) (Table 2), of which more than half received help from a personal support worker (PSW). PSWs provide personal care, ensure personal safety and provide emotional support to persons living at home. In Ontario, they typically receive 8 months of training at a college. Among those who received formal support, approximately 60% felt their needs were “very” or “extremely well” met. Seventeen caregivers received informal help (e.g., family, friends, neighbours) with caregiving activities, most of which was provided through family members but also through friends and/or neighbours. Of those receiving informal help, just over half had their needs “very” or “extremely well” met.

**Table 2:** How well caregivers’ needs were met for those who received formal help (n = 23) and informal help (n = 17).

	n (%)
**Formal Help (e.g., paid service providers, support programs, groups)**	
Minimally well	5 (21.7)
Moderately well	4 (17.4)
Very well	9 (39.1)
Extremely well	5 (21.7)
**Informal Help (e.g., family, friends, neighbours)**	
Minimally well	3 (17.6)
Moderately well	5 (29.4)
Very well	5 (29.4)
Extremely well	4 (23.5)

### Caregiver health and well-being

Table 3 summarizes results of the caregivers’ health and well-being indicators and provides ranges and cut off scores with interpretations for each indicator. Overall, caregivers reported their health as good (Mean = 3.0 [SD = 1.02]). Caregiver strain was consistently experienced by all caregivers and captured through both quantitative and qualitative data. One participant captured the intensity of the role describing caregiving as “*a position that you can’t apply for or plan for* … *there is no end in sight and that is the most debilitating part*” [ID 16]. They also experienced anxiety symptoms in the past two weeks (Mean = 9.6 [SD = 6.64]), as denoted by responses on the GAD 7 scale. Of all caregivers, 29.6% (n = 8) of caregivers had moderate symptoms of anxiety, while 22.2% (n = 6) had severe symptoms. Responses to the CESD-10 suggest that caregivers were also experiencing depressive symptoms (Mean = 11.8 [SD = 8.72]) with 55.6% (n = 15) having scores of ≥ 10 indicating depression. Scores on the MCSI were high amongst caregivers (Mean = 15.3 [SD = 7.03]). Despite the high degree of caregiver strain, caregivers were satisfied with their social supports based on the Duke Social Support Index (DSSI) (Mean = 19.3 [SD = 2.24]). However, caregivers rated their social network/social interaction somewhat less than optimal (Mean = 8.3 [SD = 3.59]) in the HL survey.

**Table 3:** Mean scores and standard deviation of instruments measuring caregiver health.

Instruments	Mean (SD)
**Overall rating of Health** (1 = poor; 5 = excellent)	3.0 (1.02)
**Modified Caregiver Strain Index (MCSI)** Scores can range from 0 [no strain] to 26 [maximum level of strain]	15.5 (7.03)
**Generalized Anxiety Disorder (GAD-7)** A score of 0–4 is considered minimal symptoms of anxiety, 5–9 is mild anxiety, 10–15 is moderate anxiety; and >15 is severe symptoms of anxiety.	9.6 (6.64)
**Center for Epidemiological Studies Short Depression Scale (CES-D 10)** Scores can range from 0 (no depression) to 30 (maximum depression score): A score ≥10 is considered depressive symptoms	11.8 (8.72)
**Duke Social Support Index (DSSI)** Score can range from 7 [limited social support] to a maximum of 21 [a high degree of social support]	19.3 (2.24)
**Quality of social network/social interaction** Score can range from 4 [limited social support] to a maximum of 12 [a high degree of social support]	8.3 (3.59)

### Characteristics of Health Links patients

Table 4 provides a summary of the characteristics of HL patients as reported by their caregivers. Patients ranged in age from 50 to 96 years (Mean = 77.7) and 59.3% were female. Results from open-ended survey questions indicated that more than one-third (n = 10; 37.0%) had cognitive impairment and/or a cardiovascular condition (n = 10; 37%), and 29.6% (n = 8) had a lung condition. Eleven caregivers (40.7%) reported that their loved one had three or more chronic conditions.

**Table 4:** Health links patient characteristics (n = 27).

**Age**			
Mean	SD	Median	Range
77.7 years	12.1	80 years	50 to 96 years
**Gender**			n (%)
Male			11 (40.7)
Female			16 (59.3)
**Health Problems** (derived from open-ended survey questions)			n (%)
Cognitive Impairments [e.g., dementia, Alzheimer’s disease, delirium]			10 (37.0)
Cardiovascular Conditions [e.g., high blood pressure, myocardial infarction, angina]			10 (37.0)
Lung Conditions [e.g., pneumonia, emphysema, pulmonary fibrosis, COPD]			8 (29.6)
Other [e.g., cancer, diabetes, arthritis, joint replacements, myelopathy, neuropathy, Parkinson’s disease]			22 (81.5)
**Number of Chronic Conditions**			n (%)
1 Chronic Condition			9 (33.3)
2 Chronic Conditions			7 (25.9)
3 or More Chronic Conditions			11 (40.7)

### HL core concepts

Themes are organized under the HL core concepts—patient and family-centred care, accessibility, continuity of care and care provider, and coordination of services. Fig 1 and S1 Table outline elements within each HL core concept. Results of the survey questions that are focused on perceived impacts of HL on caregivers’ overall physical and mental health, quality of life, as well as perceived impacts as they relate to the HL core concepts using a scale *1 (strongly disagree)* to *5 (strongly agree)* can be found in the S2 Table: Caregiver’s Perceived Impacts of HL on Themselves and the HL Patients for Whom They Provide Care (n = 27). Where relevant, survey results are integrated with qualitative findings in the following section.

#### Patient and family-centred care

**Overall satisfaction with quality of care from HL care coordinator and providers:** Almost all caregivers reported positive qualities of their HL care coordinator and that the coordinator was key to ensuring a patient and family-centred care approach to care. These qualities included being responsive, available, and linking care. One caregiver explained:

*“The HL care coordinator actually did a lot of explaining and researching… Well*, *she knew pretty much what was available in the area.”*

[ID 4]

Typical encounters with HL care coordinators consisted of home visits and phone calls. Some visits were conducted with the caregiver present, while others were not. To address patient and family needs, one caregiver described a HL coordinator who arranged for videoconferencing to support a rural patient that reduced family stress. In other instances, HL coordinators visited a patient in hospital and a caregiver’s workplace.

Most caregivers also reported positive relationships with providers (i.e., nurses, therapists, social workers, and PSWs), and found providers to be knowledgeable, experienced, friendly, and approachable. Some caregivers, however, indicated that the quality of care by some providers was poor, which was often tied to PSW performance. For example, a caregiver described a situation where the PSW failed to assist a patient to engage in physical activity to prevent atrophy because the PSW was not instructed by the supervisor to support this. In another situation, the PSW did not report patient needs to the PSW supervisor.

**Information sharing:** In the survey, caregivers strongly agreed that HL providers communicated well with the HL patient (Mean 4.2 [SD 1.19]). Qualitative findings confirmed this finding. Many caregivers experienced timely communication from service providers, such as pharmacists, meal service providers, nurses, and PSWs, to keep them up-to-date on plans. As one caregiver described,

*“I’ll be working out in my garage because [my loved one] doesn’t want me involved so much*. *And when [the therapist] leaves*, *she’ll come into the shop and talk to me for five or ten minutes if she’s got the time*, *which is very helpful to me.”*

[ID 12]

As noted in earlier examples, information sharing between PSWs and their supervisors regarding patient needs was less positive.

**Participation in care planning:** Caregivers’ perceptions related to participating in HL care planning were mixed. Most reported having a ‘hard copy’ of the care plan. About half reported that the patient was generally in charge of directing care and caregivers were involved in care plan development. As noted by one caregiver,

*“[They] informed me and [the patient] of everything… every angle or whatever way we could approach this*, *to make it better for both of us.”*

[ID 7]

Caregivers noted that HL coordinators considered the patient-family unit in developing the plan of care. This was supported by survey results which indicated that caregivers agreed that HL increased their involvement in the care decision-making for their loved one/friend (Mean 4.1 [SD 1.27)] and increased their knowledge of how to best care for their loved one/friend (Mean 3.7 [SD 1.18]). Caregivers somewhat agreed that HL helped their loved one/friend better manage his/her care. Qualitative results also showed that some caregivers were unaware of the HL care plan nor did they have a copy. A few identified that caregiver needs were not included in the care plan nor were they asked for input. As noted by a caregiver:

*“…*..*my needs were never addressed.”*

[ID 16]

**Dignity and respect:** With few exceptions, most caregivers expressed satisfaction regarding how patients’ and caregivers’ values and beliefs were respected by care coordinators and service providers. This was supported by the survey that showed that caregivers agreed that HL provided services met their loved one’s/friend’s needs (Mean = 4.2 [SD = 1.26]). One caregiver shared:

*“They were very*, *very sensitive to my needs*, *and listened to my input*.*[…].”*

[ID 5]

#### Accessibility to care

**Approachability:** Caregivers learned about HL through hospitals, home care, outpatient care, rehabilitation services, and primary care. Many caregivers explained that the HL program was easy to access given that services were generally delivered at home or by telephone. Survey results validated this indicating that caregivers agreed that HL provided services for their loved one/friend that were easy to access (Mean = 4.1 [SD = 1.30]). A few caregivers received information about community supports for themselves but were not able to access them due to: restrictive program eligibility requirements and a lack of local program availability. For some, accessing respite care was viewed as being cumbersome and too much work was needed to access support. Limited respite hours were an additional barrier.

**Availability and accommodation**: Most caregivers agreed that HL ensured there was minimal wait time for their loved one/friend to get services (Mean = 4.2 [SD = 1.26]. Despite this, some caregivers reported barriers for patients to access recommended community supports such as inconvenient appointment times and mobility challenges.

*“You must be able to*, *you know*, *walk and be ambulatory*, *let’s put it that way.”*

[ID 2]

**Affordability:** Caregivers noted that there were no direct costs to use the HL program. However, they identified that they faced out-of-pocket costs to access recommended community supports for their loved one and themselves, which made them unaffordable. Some families incurred out-of-pocket costs related to parking and transportation to access community services, medications, and equipment not covered by health insurance plans. Further, many commented on respite care limitations for caregivers in relation to available hours and cost:

*“I’m in a situation where I can’t afford it*. *I can’t afford to pay the per diem to put [HL patient] into a nursing home for a week or two so I can get a rest.”*

[ID 2]

#### Coordination of care

**Overall quality of care coordination:** Most caregivers indicated that the HL care coordinator arranged for home care services supports and developed coordinated care plans involving unregulated providers (e.g., PSWs) and regulated providers (e.g., nurses, therapists). Based on survey results, caregivers agreed that HL provided services for their loved one/friend which were well coordinated (Mean = 4.3 [SD = 1.16]). Interviews supported this as some caregivers reported that the care for their loved one was well coordinated, provided access to service providers as needed, and shared relevant information about services, costs, and contacts.

**Communicating roles and plan for care:** A few caregivers noted that everyone who was providing care knew each other’s roles and the care they each were providing. On the other hand, some caregivers identified poor communication among team members which was exacerbated by: vertical rather than horizontal reporting structures (with communication occurring through supervisors rather than directly among services providers particularly in the case of PSWs), service providers who did not speak English, and difficulties reaching providers by phone.

**Limited follow up and updates:** Some caregivers had limited or intermittent follow-up and a lack of sharing of care plan updates with service provider agencies and providers. A few caregivers described that when follow up did not occur, care was reactive rather than proactive. As one caregiver said,

“I wish that there was something that would happen before it gets to a point where he needs something more.”

[ID 15]

**Some integration of health and community support services:** Caregivers indicated that various sources of formal supports were arranged through HL. Approximately half of caregivers explained that the HL coordinator provided relevant health and social supports for patients, either through direct referrals or by providing information about services for caregivers and HL patients to follow up with on their own. One caregiver explained:

“I didn’t know that there was a program for helping people to afford walkers … it was [a] HL coordinator who set that up for us.”

[ID 2]

Examples of programs and services included foot clinics, friendly visitor programs, day programs, and transportation services for disabled clients, medical alert systems, and assistive devices programs. Some caregivers indicated that the HL care coordinator was a helpful source of information for community programs to support IADLs (e.g., meal programs, housekeeping) and in one case, financial supports. In addition, some caregivers indicated that they received suggestions about community-based supports (Alzheimer’s Society) from health care providers, such as hospital or home care coordinators.

**Coordination of care less focused on caregivers:** Care coordination was mostly focused on the patient rather than the caregiver. Survey results showed that caregivers somewhat agreed that HL increased their knowledge of where (Mean = 3.6 [SD = 1.52]) and how (Mean = 3.7 [SD = 1.49]) to get health care services for themselves. Respite services were rarely offered to caregivers. In a few cases, caregivers explained that they received no support for themselves from HL due to their desire for privacy or because they deemed that no support was necessary.

Some caregivers filled the role of a care coordinator to ensure continuity and address the gaps in coordination, despite having formal HL care coordinators. A few caregivers described being the de facto care coordinators, primarily responsible for organizing care, following up with various services, and acting as the main source of patient information.

*“[Service providers] provide you the numbers and the offices to call and coordinate*, *and you do the coordination.”*

[ID 5]

In a few cases, caregivers found support services for house cleaning and meal services, and caregiver support groups. Caregivers shared their need for better system navigation support as some were given inadequate information to follow up with suggested services.

#### Continuity of care

**Continuity of care providers:** Survey results indicated that caregivers agreed that HL ensured that the same people took care of their loved one/friend (Mean = 4.1 [SD = 1.19]. Half of caregivers in qualitative interviews reported experiencing continuity in service providers, including nurses, therapists, and PSWs. Caregivers expressed that this gave them relief and provided patients with needed support and companionship, ensuring relational continuity. Inconsistency in service providers, when experienced, was related to home care coordinator changes in staff assignments, having different staff covering weekends, and movement or attrition of staff. Changes in service providers led to increased stress by HL patients and caregivers.

*“When the other people come in*, *sometimes they have to show them how to use the shower*, *have to show them where the clothes are*. *And this happens every week*, *almost*, *this rotating PSW scenario*. *So*, *that causes stress*, *and that means I can’t leave the home.”*

[ID 15]

One family had ongoing PSW personnel changes, which was driven by workers’ challenges in managing the patient who was suffering from dementia. Changes in care providers were so disruptive in this situation that the caregiver decided to cancel the service despite badly needing support. Caregivers appreciated when providers maintained regular schedules for visits and informed them ahead of time of any scheduling changes. A caregiver commented on the importance of communicating any changes in providers:

*“And if she can’t come on time*, *she’ll call*. *[Good*.*] And she always tells my mother if she’s not going to be there in a few days.”*

[ID25]

**Continuity of care provision:** Caregivers described mixed experiences in terms of consistency in the care provided. In the absence of caregiver continuity, some participants reported that shared documentation left in the home was a good strategy to support informational continuity (i.e., where information on prior events is used to give care that is appropriate to the patient’s current circumstance). One caregiver explained:

*“[Service providers]* *always have this health care binder […] It was just a patient information binder that they always had with them and [they] would make their records*, *and then they could all refer back.”*

[ID 23]

Survey respondents agreed that HL ensured that there was minimal disruption in care provided to their loved one/friend (Mean = 4.2 [SD = 1.17]) [1 (strongly disagree) to 5 (strongly agree)]. Some caregivers indicated that there was a good degree of information sharing among service providers either through documentation or overlapping visits which happened by coincidence. They perceived that this resulted in providers working together as a team and improved understanding of each other’s roles and scope. In contrast, many other caregivers identified challenges associated with consistency of care provision, related to the replacement of PSWs who were unfamiliar with the care to be provided, or were inadequately trained to manage care complexities. One caregiver explained that there were changes in “*duties*” that the PSW was permitted to conduct (e.g., help with laundry): This was related to funding cut-backs and reduced PSW time.

#### Impacts of health links

Impacts were measured on a scale of 1 (strongly disagree) to 5 (strongly agree). Survey results indicated that caregivers were very satisfied with HL overall (Mean = 4.1 [SD = 1.23]) and with HL as a service for their loved one/ friend (Mean = 4.0 [SD = 1.22]). However, caregivers were indifferent or were “somewhat dissatisfied” with HL’s impact on them as a caregiver or on the care recipient. Caregivers “neither agreed nor disagreed” when asked if HL had improved their quality of life or improved their mental health (Mean = 3.4 [SD = 1.25] and Mean = 3.0 [SD = 1.21] respectively). Caregivers disagreed that HL had helped improve their physical health but on average somewhat agreed that it helped their loved one better manage their care (Mean = 2.8 [SD = 1.30] and Mean = 3.7 [SD = 1.18] respectively). Qualitative results supported these results to some degree. Some caregivers felt indifferent about the impact of the HL program for themselves and their loved one. When asked if caregiver needs were addressed by HL a caregiver explained:

“*As far as the work that I had to do*, *no not really*. *I was sort of left to my own devices to*, *you know*, *do what needed to be done.”*

[ID23]

A few did not take advantage of what was offered or felt that they did not need the support. However, most caregivers were generally positive about the impacts of the HL program. Some caregivers explained that patients experienced improvements in the health and ADLs which made life easier. Others spoke about having increased responsiveness to address patients’ needs. For example, there were referrals to needed services and access to services that were previously unknown. There were individual instances mentioned that a loved one: was more independent, had companionship from consistent and caring PSWs, and was still living at home.

#### Recommendations for improvements to the Health Links program

Caregivers recommended improvements to the HL program, suggesting that the HL care coordinators could provide more follow-up regarding uptake of recommended services and health status; conduct occasional reassessments; offer more support in relation to IADLs, such as housekeeping; and coordinate more respite hours to provide relief for caregivers. Most caregivers suggested that information related to community programs and supports needs to be easy to access. Many preferred an online or a ‘hard copy’ resource that emphasized tailored local information including program details that the caregiver could review when needed. Given the complexity of caregiving, information about available community programs and services needs to be presented in an organized way early in the HL contact, easily retrievable when required, and revisited. One caregiver recommended:

*“I’d like to have [the information] given in an easy to access form*, *‘cause I use a computer*. *I could access it on a database as opposed to having disparate pieces of paper in a folder that I may or may not have time to look at*, *even though they mention everything in it at the beginning*. *Once they’re gone and I’m carrying on with my duties here*, *it’s forgotten*. *A re-emphasis on a revisiting of the services might have helped me*. *I think repeating it over and over because I’m afraid most of the time*. *I know what the things that they gave and said*, *but maybe the paper was placed somewhere else.”*

[ID15]

To improve patient experiences and outcomes, caregivers highlighted a need to improve care to address physical needs of patients as well as social and emotional needs, particularly for those identified as being lonely and isolated. Skill development for PSWs on how to manage and work with patients with dementia was also identified as an urgent need.

## Discussion

This study is unique in that it examined family and friend caregivers’ perceptions of a provincial program aimed at addressing the highest users of the health care system. Caregivers of patients in the HL program took on multiple responsibilities, including assisting with IADLs and less frequently ADLs. As supported by others [8, 16, 34–36], caregivers identified that this role greatly impacted their lives. Caregiving in the current study was associated with high levels of caregiver strain, depressive symptoms, mild anxiety, and less than optimal social networks and interactions. This mirrors results from other studies of caregivers of patients with complex health issues.[9, 10, 37] Caregivers ranged in age, education levels and number of years of caregiving experience. The heterogeneity of caregivers as seen in this study was also reported by others.[16, 35] Given this and the perceived negative impact of caregiving on health and wellbeing, tailored interventions are needed to support caregivers.

Overall, caregivers were satisfied with the HL program in terms of support for the patient but not for their own needs. In relation to patient- and family-centred care, most, but not all caregivers had a copy of the patient’s care plan, which is an essential component of the HL program. Although care plans were completed, they were not always revisited or addressed in subsequent visits. Caregivers indicated that HL was primarily focused on patients with relatively little attention to caregivers’ needs. Caregivers were typically not incorporated in the coordinated care plans and some were not asked for their input. Patient- and family-centred care must involve collaboration with patients, families and care providers.[38, 39] Care plans for patients with complex or multiple chronic conditions should consider caregivers’ needs and incorporate their input given the impact of caregiving on their lives.

With respect to care coordination, most caregivers agreed that service providers and HL care coordinators were generally responsive, aligned care to HL patients’ needs, and were respectful of patients’ values and beliefs. Some caregivers expressed that they acted as the care coordinators for their loved ones, which was vital to ensure continuity of care and manage problems resulting from poor care coordination by some HL coordinators. Care coordination tended to be reactive rather than proactive causing lags in action taken to address issues as they were developing. Caregivers wanted more frequent health status reviews through patient reassessments so that care would be more proactive. This points to the value of regular follow up and review of care plans.

Even though caregivers in the current study reported that care was delivered by knowledgeable and dependable service providers, they felt that communication among the care team was less than adequate. In some cases, this was related to vertical rather than horizontal reporting structures, such as communication through coordinators and supervisors rather than directly among service providers. Further, there were mixed perceptions related to continuity of care providers. Not surprisingly, those with continuity of care providers reported that patients received needed companionship and support and reduced caregiver stress. While a lack of provider continuity resulted in anxiety and stress in both patients and caregivers. Lack of provider continuity was linked to inconsistency in care provision, particularly in relation to replacements of PSWs who were unfamiliar with the patient and care to be provided. This was exacerbated by poor communication among service providers and supervisors. One potential solution to address the problems of continuity of care providers and communication challenges is the application of self-managed integrated teams.

The Dutch Buurtzorg model focused on self-managed teams and integrated care [40] offers a potential strategy to address many of the problems highlighted by caregivers of patients in the HL model. The model has shown early positive results that address team communication problems, do away with the top-down system of care, and address continuity of care issues.[41, 42] Some core components of the Buurtzorg model include robust team communication involving weekly team meetings, frequent shared casual contacts, nurses providing comprehensive holistic care (rather than multiple providers), relationship-based practice, client empowerment, and technology supports. A UK case study of a pilot implementation of an adapted Buurtzorg model delivering care by a self-managing neighbourhood nursing team to patients with multiple chronic conditions resulted in more proactive care with multiple follow ups, and was found to be feasible and acceptable to patients, family carers, and providers.[41] Although further comparative and longitudinal research is needed, the HL model might benefit from integration of the self-managing team feature of the Buurtzorg model to overcome some of the challenges identified in the current study.

Caregivers in the current study appreciated that HL was accessible as it was provided through home visits and telephone calls. Despite this support, HL caregivers described challenges in accessing needed products and services for themselves and their loved ones. Accessibility barriers included program eligibility restrictions, out of pockets costs for medication and equipment costs not covered by government programs, poor availability of services, transportation costs to access community programs and services, and cumbersome processes to arrange for respite. Specifically, caregivers asked for more coordination, follow up, and financial support with respect to caregiver respite services. As others have found [34, 43], there is a need for caregivers to also have access to information on where and how to find recommended health and community services to support them and their loved one.

Given the results of this study, the HL program and similar programs should consider the needs of caregivers. Table 5 summarizes recommendations to address the needs of caregivers of patients with complex needs. It behooves policy-makers to move to a system where coordination involves caregivers in the circle of care as well as considers caregivers’ needs.[44, 45] There is an economic cost associated with not providing caregivers support given their important role. Mechanisms are needed to facilitate care coordination that support continuity of care and effective communication among team members including health and social service providers that ultimately results in better care for the patient and reduces caregiver stress.[46, 47] Care coordination needs to incorporate navigation to community support services to support caregivers as well as patients. Given the burden of caregiving, further research is needed to explore enhanced models of care that ensures support to caregivers of high users of the health care system in programs such as HL.

**Table 5:** Recommendations for caring for caregivers of patients who have complex care needs.

Implement tailored interventions to support caregivers in addressing their individual needs in addition to those of the patient.
Consider caregivers’ needs and incorporate caregiver input into care plans given the impact of caregiving on their lives.
Follow up and review the care plan regularly to support proactive versus reactive care.
Consider the implementation of self-managed neighbourhood teams involving weekly team meetings and technology to support coordination and overcome problems of continuity in care providers and team communication challenges.
Provide coordination, follow up, and financial support for caregiver respite.
Provide system navigation and an easily retrievable hard copy list of relevant health and community services to support patients and their caregivers.
Offer more patient supports for IADLs to reduce caregiver burden.

### Study strengths and limitations

This study used several reliable and valid tools and a survey that was developed and pretested by the team to describe the characteristics of caregivers and HL patients and caregiver’s perceptions of the HL program. Although this study included perspectives from a wide range of caregivers representing 3 of 14 regional health authorities and 6 HL programs, these sites may not be representative of the province. Given the small sample size, results for the research question related to characteristics of caregivers of patients enrolled in HL program are not generalizable and requires more rigorous study to draw any conclusions. The available sample for this study was directly linked to the success of our university partner who was responsible for recruiting HL patients, as this was the only way that we could identify HL caregivers for the study. Despite our partners’ strong attempts at recruiting from multiple HL programs, there were administrative challenges in obtaining HL’s patient names from some HL programs that limited the ability to reach patients and in turn, their caregivers. Working in close collaboration not only with provincial but regional and/or local government authorities is key to recruiting patients and caregivers in research. Recruiting caregivers has been identified as challenging given the multiple demands on their time, the potential for social isolation, and a lack of trust in institutions [48]. Engaging in research that involves patient-caregiver dyads may be one way to better gain access to them. Despite our recruitment challenges, this study obtained a diverse sample of caregivers receiving care from different types of providers. In addition, quantitative results validated qualitative findings strengthening the rigor of this research through methodological triangulation.

The HL program is complex in that it supports links to community-based health and social services to address the complex needs of patients and their caregivers. Given this, it is not surprising that some caregivers had challenges differentiating the HL program from care provided from other programs and services. Finally, given that this study was a focused on caregiver perceptions, we can only report on perceived impacts. Further study of caregivers of high systems users would be valuable to gain a stronger understanding of the challenges commonly faced and supports that help them. Future studies should examine associations between factors, or variables in this study, to determine the correlates of positive caregiver outcomes which can inform the development of interventions for caregivers. Following this, longitudinal research methods should examine the implementation and effectiveness of new models of care for high system users.

## Conclusions

Caregivers experienced significant strain, anxiety and depressive symptoms indicating that more attention needs to be paid to their needs. Their perceptions varied based on the care provided to HL patients by care coordinators and service providers. Caregivers appreciated accessible, consistent, coordinated, patient and family-centred and team-based approaches to care. Policy and decision-makers are urged to ensure that programs aimed at high system users with multiple chronic conditions include these core concepts. To enable a truly family-centred approach, such programs cannot ignore caregivers’ needs. Future research is needed to explore the effects of enhanced models of care for patients with complex needs that incorporates care for caregivers.

## Supporting information

S1 File Health Links Caregiver survey. (DOCX)

S2 File Health Links Caregiver Evaluation Study Interview Guide. (DOCX)

S1 Table Health Links core concepts. (DOCX)

S2 Table Caregiver perceived impacts of Health Links on themselves and HL patients for whom they Provide Care (n = 27). (DOCX)
