# Supplementary material for: An evaluation study of caregiver perceptions of the Ontario’s Health Links program
Source: PLoS One. 2020 Feb 27;15(2):e0229579. doi: 10.1371/journal.pone.0229579 (PMC7046224; doi:10.1371/journal.pone.0229579)
Supplement: S1 File — (DOCX) [file pone.0229579.s001.docx]

## **S1 File**

## **H****ealth Links Evaluation: Caregiver Survey**

- You are being asked to participate in a study and complete this survey because you have been identified as a **primary caregiver for a person who is enrolled in Health Links**.
- **Health Links** helps people to find the care they need. It is designed to help people who have more than one type of health concern. People enrolled in Health Links are *linked* to a contact person or helper; this is someone who knows them and their medical history. Health Links also works behind the scenes to help doctors, nurses, and community organizations work together as a team. In your community, Health Links services are provided by [Name of Organization].
- A primary caregiver is a person who helps someone living with multiple health concerns. A caregiver can help in many different ways. They do tasks and help with managing day-to-day activities. Caregivers can give emotional support. They may help with finances or offer other types of support.
- If you agree to participate in this study, a research assistant will ask the survey questions over the telephone.
- The survey will take approximately 30 -45 minutes.
- You do not have to answer any questions you do not want to. Your participation is completely voluntary!

**Thank you for your interest in this study!**

Participant ID #: ____________________

**Ca****regiver Role**

**You are a family member or friend of a person who is enrolled in Health Links. Please tell me more about your relationship with this person and the support you provide.**

1. What is your relationship to the person enrolled in Health Links?

I am a...

- - Spouse/partner
  - Sibling (brother or sister)
  - Son or daughter
  - Son-in law or daughter-in-law
  - Grandson or granddaughter
  - Nephew or Niece
  - Friend
  - Neighbour
  - Other (please specify):

1. Do you live with the person you are caring for?
   - Yes
   - No
2. Tell me about your caregiver role?
3. What kind of assistance do you provide to your loved one/friend? Check all that apply.

I help my loved one/friend with:

- - Bathing
  - Dressing
  - Helping to use the Toilet
  - Managing soiling from Stool or Urine
  - Food Preparation
  - Feeding
  - Taking their Medication
  - Using the Telephone
  - Housekeeping
  - Laundry
  - Finances
  - Shopping
  - Transportation
  - Other (please specify): ________________________________________________

5. How long have you been providing care to [Name of Care Recipient]?

________ **months**

1. On average, how many hours **per week** do you spend providing care to [Name of Care Recipient]?

_________ **hours**

7 a). Do **you** receive **informal help** with your caregiving activities? (For example, help from family, friends and/or neighbours).

- - Yes
  - No

**If NO …. GO to Question 8**

1. How well does this **informal** help meet your needs? Please select one response.

- Not at all
- Minimally well
- Moderately well
- Very well
- Extremely

8.a) Do **you** receive **formal help** for your caregiving activities? (For example, help from paid service providers, a support program or a group.)

- - Yes
  - No

**If NO…Go to Question 9**

b) How well does this formal help meet your needs? Please select one response.

- Not at all
- Minimally well
- Moderately well
- Very well
- Extremely

9. Do you receive palliative care services for [Name of Care Recipient]?

- - Yes
  - No

**If YES ….** From whom: (please specify): ______________________________________________

**Your Experience and Satisfaction with Health Links**

Your loved one/friend is enrolled in Health Links. Health Links helps people find the care they need. In your community, Health Links services are provided by [Name of Organization].

We are interested in your feedback on **Health Links** and how well it helps **you and** **your loved one/friend**. Please tell us how you feel about Health Links by rating **how much** you **disagree or agree** with the following statements.

First, **think about yourself** as a **Caregiver….**

| **As a caregiver, Health Links has helped….** | Strongly Disagree | SomewhatDisagree | Neither Agree nor Disagree | Somewhat Agree | Strongly Agree |
| --- | --- | --- | --- | --- | --- |
| 1. …increase my knowledge of **where** to get health care services for myself. | 1 | 2 | 3 | 4 | 5 |
| 1. ….increase my knowledge of **how** to get health care services for myself. | 1 | 2 | 3 | 4 | 5 |
| 1. …improve my physical health. | 1 | 2 | 3 | 4 | 5 |
| 1. … improve my mental health. | 1 | 2 | 3 | 4 | 5 |
| 1. …improve my quality of life. | 1 | 2 | 3 | 4 | 5 |
| 1. ...increase my knowledge of how to best care for my loved one/friend. | 1 | 2 | 3 | 4 | 5 |
| 1. …increase my involvement in the care decision-making for my loved one/friend. | 1 | 2 | 3 | 4 | 5 |

Now, think about your **loved one/friend** to **whom you provide care**….

| **Health Links has….** | Strongly Disagree | Somewhat Disagree | Neither Agree nor Disagree | Somewhat Agree | Strongly Agree |
| --- | --- | --- | --- | --- | --- |
| 1. …ensured there is minimal wait time for my loved one/friend to get services. | 1 | 2 | 3 | 4 | 5 |
| 1. ….ensured there is minimal disruption in the care provided to my loved one/friend. | 1 | 2 | 3 | 4 | 5 |
| 1. …ensured the same people take care of my loved one/friend. | 1 | 2 | 3 | 4 | 5 |
| 1. ...communicated well with my loved one/friend. | 1 | 2 | 3 | 4 | 5 |
| 1. ….helped my loved one/friend better manage his/her own care. | 1 | 2 | 3 | 4 | 5 |
| 1. ….provided services that meets my loved one’s/friend’s needs. | 1 | 2 | 3 | 4 | 5 |
| 1. …provided services for my loved one/friend that are easy to access. | 1 | 2 | 3 | 4 | 5 |
| 1. …provided services for my loved   one/friend that are well  coordinated. | 1 | 2 | 3 | 4 | 5 |

Thinking about **Health Links** as a whole….

1. Overall, how **satisfied** are you with **Health Links** as **a caregiver**?
   - Very Dissatisfied
   - Somewhat Dissatisfied
   - Neither Satisfied or Dissatisfied
   - Somewhat Satisfied
   - Very Satisfied
2. Overall, how **satisfied** are you with **Health Links** for **your loved one/friend**?
   - Very Dissatisfied
   - Somewhat Dissatisfied
   - Neither Satisfied or Dissatisfied
   - Somewhat Satisfied
   - Very Satisfied

**Your Health and Well-Being (General)**

We would like to learn a little more about your health and well-being as a caregiver.

1. Which of the following best describes your health? (Please select one response).
   - Poor
   - Fair
   - Good
   - Very Good
   - Excellent

2.a). In the **past 6 months,** have you had to go to a health care provider to get help regarding your health?

- - Yes
  - No

If **Yes**: How many times: ______

2.b). Who did you see?

- - Family doctor
  - Doctor in urgent care
  - Specialist (Doctor)
  - Physiotherapist
  - Occupational Therapist
  - Chiropractor
  - Naturopath
  - Psychologist
  - Social Worker
  - Other (please specify): _______________________________________________________

1. In the **past 6 months**, have you had to go to an emergency department to get help for yourself?
   - Yes
   - No

If **Yes**: How many times: ______

1. In the past 6 months, have you been hospitalized?
   - Yes
   - No

If **Yes**: How many **days**: ______

**Yo****ur Health and Well-Being (Modified Caregiver Strain Index)**

Below is a list of things that other caregivers have found to be difficult. Please check the box that applies to you. We have included some examples that are common caregiver experiences to help you think about each item. Your situation may be slightly different, but the item could still apply.

|  | **Yes, on a regular basis** | **Yes, sometimes** | **No** |
| --- | --- | --- | --- |
| 1. My sleep is disturbed. *(For example: the person I care for is in and out of bed or wanders around at night.)* | 2 | 1 | 0 |
| 2. Caregiving is inconvenient. *(For example: helping takes so much time or it’s a long drive over to help.)* | 2 | 1 | 0 |
| 3. Caregiving is a physical strain. *(For example: lifting in and out of a chair; effort or concentration is required.)* | 2 | 1 | 0 |
| 4. Caregiving is confining. *(For example: helping restricts free time or I cannot go visiting.)* | 2 | 1 | 0 |
| 5. There have been family adjustments. *(For example: helping has disrupted my routine; there has been no privacy.)* | 2 | 1 | 0 |
| 6. There have been changes in personal plans. *(For example: I had to turn down a job; I could not go on vacation.)* | 2 | 1 | 0 |
| 7. There have been other demands on my time. *(For example: other family members need me.)* | 2 | 1 | 0 |
| 8. There have been emotional adjustments. *(For example: severe arguments about caregiving.)* | 2 | 1 | 0 |
| 9. Some behaviour is upsetting. *(For example: incontinence; the person cared for has trouble remembering things; or the person I care for accuses people of taking things.)* | 2 | 1 | 0 |
| 10. It is upsetting to find the person I care for has changed so much from his/her former self. *(For example, he/she is a different person than he/she used to be.)* | 2 | 1 | 0 |
| 11. There have been work adjustments. *(For example: I have to take time off for caregiving duties.)* | 2 | 1 | 0 |
| 12. Caregiving is a financial strain. | 2 | 1 | 0 |
| 13. I feel completely overwhelmed. *(For example: I worry about the person I care for; I have concerns about how I will manage.)* | 2 | 1 | 0 |

**Yo****ur Health and Well-Being (CES-D 10: Center for Epidemiological Studies Short Depression Scale)**

Below is a list of the ways you might have felt or behaved recently. For each of the following statements, please *check the box which best describes how often you have felt this way* ***during the last week.***

| **During the last week:** | Rarely or none of the time  (less than 1 day) | Some or a little of the time (1-2 days) daysdays) | Occasionally or moderate (3-4 days) | Most or all of the time (5-7 days) |
| --- | --- | --- | --- | --- |
| 1. I was bothered by things that usually don’t bother me. | 0 | 1 | 2 | 3 |
| 2. I had trouble keeping my mind on what I was doing. | 0 | 1 | 2 | 3 |
| 3. I felt depressed. | 0 | 1 | 2 | 3 |
| 4. I felt that everything I did was an effort. | 0 | 1 | 2 | 3 |
| 5. I felt hopeful about the future. | 0 | 1 | 2 | 3 |
| 6. I felt fearful. | 0 | 1 | 2 | 3 |
| 7. My sleep was restless. | 0 | 1 | 2 | 3 |
| 8. I was happy. | 0 | 1 | 2 | 3 |
| 9. I felt lonely. | 0 | 1 | 2 | 3 |
| 10. I could not get *“going”*. | 0 | 1 | 2 | 3 |

**Yo****ur Health and Well-Being (GAD – 7: Anxiety)**

Below is a list of how people may feel. For each of the following statements, please *check the box which best describes how often you have felt this way* ***during the last two weeks.***

| **Over the last two weeks, how often have you been bothered by the following problems?** | **Not at all** | **Several days** | **More than half the days** | **Nearly every day** |
| --- | --- | --- | --- | --- |
| 1. Feeling nervous, anxious, or on edge | 0 | 1 | 2 | 3 |
| 2. Not being able to sleep or control worrying | 0 | 1 | 2 | 3 |
| 3. Worrying too much about different things | 0 | 1 | 2 | 3 |
| 4. Trouble relaxing | 0 | 1 | 2 | 3 |
| 5. Being so restless that it is hard to sit still | 0 | 1 | 2 | 3 |
| 6. Becoming easily annoyed or irritable | 0 | 1 | 2 | 3 |
| 7. Feeling afraid, as if something awful might happen | 0 | 1 | 2 | 3 |

If you checked off any problems, how difficult have these made it for you to do your work, take

care of things at home, or get along with other people?

- - Not difficult at all
  - Somewhat Difficult
  - Very Difficult
  - Extremely Difficult

**Your Health and Well-Being (Duke Index of Social Support)**

**These questions will help us understand your relationships with friends and family and who supports you.**

1. Do you feel you have a definite role in the family and among friends?

- - Hardly ever
  - Some of the time
  - Most of the time
  - No Answer

1. Do family and friends understand you?
   - Hardly ever
   - Some of the time
   - Most of the time
   - No Answer
2. Do you feel useful to family and friends?
   - Hardly ever
   - Some of the time
   - Most of the Time
   - No Answer
3. Do you feel listened to by family and friends?
   - Hardly ever
   - Some of the time
   - Most of the time
   - No Answer

5. Do you know what’s happening with family and friends?

- - Hardly ever
  - Some of the time
  - Most of the time
  - No Answer

6. Can you talk about your deepest problems? [with at least one of your family or friends.]

- - Hardly ever
  - Some of the time
  - Most of the time
  - No Answer

7. How satisfied are you with relationships with family and friends?

- - Hardly ever
  - Some of the time
  - Most of the time
  - No Answer

8. Number of family members within 1 hour that you can depend on or feel close to: _____

- - No answer

9. Number of times this past week spent with someone not living with you: _____

- - No answer

10. Number of times in past week talked with friends/relatives on the telephone: _____

- - No answer

11. Number of times in the past week attended meetings of clubs, religious groups, or other groups that you belong to (other than work): _____

- - No answer

**Ca****regiver Demographic Form**

**Please tell us about yourself:**

1. What is your year of birth? (yyyy)
2. Gender: Male Female Transgendered
3. What is your marital status?
   - Single Married Widowed Divorced/separated Other (specify): _________
4. Are you aboriginal?
   - No Yes

If yes, please specify:

- - First Nations (North American Indian) - Status
  - First Nations (North American Indian) - Non-status
  - Métis
  - Inuit

1. Were you born in Canada?
   - Yes
   - No

If No: How long have you lived in Canada: ______ Years

1. What is your highest level of education?
   - No Schooling
   - Elementary school (8th grade / less)
   - No high school diploma
   - High school diploma (or GED)
   - Registered Apprenticeship or other trades certificate or diploma
   - College, GCEP, or other non-university certificate or diploma
   - Bachelor’s degree
   - Master’s degree
   - Doctorate degree
2. Do you work outside of the home?
   - Yes
   - No

If yes: On average, how many hours per week do you work outside the home?

__________ hours per week

1. Do you have any dependents?

Yes  **If yes**, please specify: How many? What are their ages?___________

- - No

1. Are you receiving social services?
   - Yes
   - No
2. Are you receiving Old Age Security?
   - Yes
   - No
3. *Do you have to use your own money to support the caregiving of your loved one/ friend?*
   - Yes
   - No

**C****are Recipient Demographic Form**

**Please tell us about the person you are caring for:**

1. Age: _______________ Years

1. Gender: Male Female Transgendered
2. What health problem(s) does your loved one/friend have?

We very much value the information that you have already provided today through this survey.  In addition to this survey, we are very interested in exploring caregivers’ experiences in more depth. We will do this by interviewing a small group of caregivers. It would help our team get a deeper understanding of caregivers’ experiences and the impact of Health Links. The final survey and interview results will be combined to make recommendations for improvements to Health Links.

**Would you be willing to participate in a telephone or in-person interview at a time convenient to you? The interview will last approximately one hour either by telephone or if you prefer in-person.**

NO - Thank you very much for your valuable feedback today.

YES – Thank you very much. Could you suggest a time for us to contact you for the interview?

**Thank you for completing this survey!**
