# Supplementary material for: An evaluation study of caregiver perceptions of the Ontario’s Health Links program
Source: PLoS One. 2020 Feb 27;15(2):e0229579. doi: 10.1371/journal.pone.0229579 (PMC7046224; doi:10.1371/journal.pone.0229579)
Supplement: S2 File — (DOCX) [file pone.0229579.s002.docx]

## **S2 File**

## **Health Links Caregiver Evaluation Study: Interview Guide**

You are being asked to participate in this interview because you are a primary caregiver for a person who is enrolled in the Ontario Ministry of Health and Long-term Care’s “Health Links” program. Health Links helps people to find the care they need. It is designed to help people who have more than one type of health concern. Health Links works in different ways. Patients enrolled in Health Links are *linked* to a contact person or care coordinator; this is someone who knows them and their medical history. Health Links also works behind the scenes to help doctors, nurses, and community organizations work together as a team. In your community, Health Links services are provided by (*state the name of the local Health Links*).

[*INSERT HERE a brief description of the Health Links programming provided in the respondent’s geographic area. NOTE: Each participating Health Links will be asked to provide a 3 sentence statement that summarizes of the types of services they provide to clients in their area*.]

We want to know about your experiences with Health Links. We also want to know what you think the impact has been of Health Links on you and (*state the care recipient’s name*).

- 1. **What is your relationship with (*state the care recipient’s name*)?**

Probe: Husband, daughter/son, father, mother, neighbour, etc.

- 1. **Thinking about your role as a caregiver of a Health Links patient, what worked well for you and did not work well for you in the Health Links program**?
  2. **Support can be many different things. What kind of supports has Health Links given to you?** Probe: Think about supports to help you complete normal daily activities (Physical health), participate in social activities, or cope with stress (Mental health)?]
  3. **How do you as a caregiver and** (*state the care recipient’s name*) **benefit from health Links?**
  4. **What do you think are limitations of Health Links for you as a caregiver and for**

**(*state the care recipient’s name*)?**

**Patient and Family Centred Care**

1. **Describe a typical phone call, or a typical meeting with, Health Links providers, or the care coordinator that included you. What was your experience like in working with the team?**
2. **How would you describe the quality of relationships among members of the team, you and**

(*state the care recipient’s name*)**?**

1. **In the Health Links program, one key activity is creating a plan of care which is written down on paper for** (*state the care recipient’s name*)**. This is so that everyone knows the plan. Tell me about your experience in the care planning process and what did you think about it?**

PROBES

1. What did you know about the care plan and how did you find out about it?
2. What kind of information was shared with you about ways to address (*state the care recipient’s name*) needs, for example, information about programs available in the community that could help?
3. How sensitive were providers to your values and beliefs when planning care for (*state the care recipient’s name*) and you as a caregiver?
4. How sensitive were providers to (*state the care recipient’s name*) values and beliefs when planning his/her care?
5. During interactions with the Health Links program, in what ways were you and (*state the care recipient’s name*) involved in the developing the care plan?
6. How were your needs as a caregiver addressed in the plan of care?
7. **Thinking about your interactions with Health Links providers, we you involved in any other way in making decisions with the team and** *(state the care recipient’s name)***? If so, what was this experience like?**

**Accessibility to Care**

1. **How did you learn about the Health Links program and how did (state the care recipient’s name) get enrolled? What was the process like?**
2. **How well did the program take into account** **(state the care recipient’s name) and your personal values and beliefs about health and wellness?**
3. **How easy was it to get to and use the Health Links program (e.g., on a bus route; timely, ease in scheduling appointments?**
4. **What kind of out of pocket costs did you have, if any, for caregiving as it relates to Health Links? (e.g., transportation costs for new programs offered, time off work)**
5. **How well did the Health Links program address/meet your needs as a caregiver and**

(*state the care recipient’s name*) **needs? Please explain.**

**Continuity of care and continuity of provider**

1. **There should be consistency in care over time. For example, information that is shared with you and** (*state the care recipient’s name*) **should not conflicting across difference providers. What has been your experience with consistency in care in the Health Links program?**
2. **For good quality of care, it is important that there is consistency in the person/s who provide care over time. What has been your experience with consistency in the people who are providing you and** (*state the care recipient’s name*) **with care?**

**Coordination of Care**

One of the main roles of Health Links is to coordinate care for the patient which may include their caregiver. When many providers are involved in order to coordinate care well everyone should:

1. understand each other’s roles.
2. understand the resources that each provider can offer.
3. share information to support the patient care to make it easy to get all the care that is needed.
4. **What are your thoughts about how care was coordinated for (state the care recipient’s name) and you as a caregiver?**

Probes: Links to health and community services are made and there is follow up to ensure uptake of services**.**

1. **How well do you think all of** **(state the care recipient’s name) needs were addressed including health and social needs?**
2. **How well do you think all of your caregiving needs were addressed including your health and social needs?**

**Outcomes**

1. **How has Health Links made your life easier or better as a caregiver?**

Probes: Links to services; more knowledge about caregiving; more coordinated plan of care for the patient; feelings about caregiving improved.

1. **How has Health Links made your life more difficult as a caregiver?**

Probes: Stress, added time needed for provision of care, communication with health care providers; feelings about caregiving ; links to and use of health care and social services for you

1. **How has Health Links made** (*state the care recipient’s name*) **life easier or better?**
2. **How has Health Links made** (*state the care recipient’s name*) **life more difficult?**
3. **What changes or improvement can be made to Health Links?**
4. **Is there anything else you think is important for us to know?**
