# Supplementary material for: An evaluation study of caregiver perceptions of the Ontario’s Health Links program
Source: PLoS One. 2020 Feb 27;15(2):e0229579. doi: 10.1371/journal.pone.0229579 (PMC7046224; doi:10.1371/journal.pone.0229579)
Supplement: S1 Table — (DOCX) [file pone.0229579.s003.docx]

**S1: Table**

**Health Links Core Concepts**

Patient and Family-Centred Care includes the characteristics in the following table. Caregivers are considered ‘family’ in this context.

| **Patient and Family-Centred Care** |
| --- |
| 1. “Dignity and Respect. Health care practitioners listen to and honor patient and family perspectives and choices. Patient and family knowledge, values, beliefs, and cultural backgrounds are incorporated into the planning and delivery of care.” [1] p.1 |
| 1. “Information Sharing. Health care practitioners communicate and share complete and unbiased information with patients and families in ways that are affirming and useful. Patients and families receive timely, complete, and accurate information in order to effectively participate in care and decision-making.” [1] p.1 |
| 1. “Participation. Patients and families are encouraged and supported in participating in care and decision-making at the level they choose.” [1] p.1   *Shared decision-making* characteristics [2] include:   - 1. “Shared decision-making involves at least two participants -- the physician and patient.” [2] p. 685   2. “Both parties (physicians and patients) take steps to participate in the process of treatment decision-making.” [2] p. 685   3. “Information sharing is a prerequisite to shared decision-making.” [2] p. 687   4. “A treatment decision is made and both parties agree to the decision.” [2] p. 688 |
| 4. “Collaboration**.** Patients, families, health care practitioners, and leaders collaborate in policy and program development, implementation, and evaluation; in health care facility design; and in professional education, as well as in the delivery of care.” [1] p.1 |

**Accessibility to Care** includes the characteristics noted in the following table.

| **Accessibility to Care** |
| --- |
| “Approachability: relates to the fact that people facing health needs can actually identify that some form of services exists, can be reached, and have an impact on the health of the individual.” [3] p.5 |
| “Acceptability: relates to cultural and social factors determining the possibility for people to accept the aspects of the service (e.g. the sex or social group of providers, the beliefs associated to systems of medicine) and the judged appropriateness for the persons to seek care.” [3] p.5 |
| “Availability and accommodation: refers to the fact that health services (either the physical space or those working in health care roles) can be reached both physically and in a timely manner.” [3] p.6 |
| “Affordability: reflects the economic capacity for people to spend resources and time to use appropriate services. It results from direct prices of services and related expenses in addition to opportunity costs related to loss of income.” [3] p.6 |
| “Appropriateness: denotes the fit between services and clients need, its timeliness, the amount of care spent in assessing health problems and determining the correct treatment and the technical and interpersonal quality of the services provided.” [3] p.6 |

**Continuity of Care and Care Provider** includes the characteristics noted in the following table.

| **Continuity of Care and Care Provider** |
| --- |
| Continuity of care refers to “How one patient experiences care over time as coherent and linked: this is the result of good information flow, good interpersonal skills, and good coordination of care.” [4](p. i). “Informational continuity means that information on prior events is used to give care that is appropriate to the patient's current circumstance. Relational continuity recognizes the importance of knowledge of the patient as a person; an ongoing relationship between patients and providers is the undergirding that connects care over time and bridges discontinuous events.” [4] (p. i). “Management continuity ensures that care received from different providers is connected in a coherent way. Management continuity is usually focused on specific, often chronic, health problems” [4] (p. i). |
| Continuity in care provider is an enabler of care continuity and refers to “…the patient's experience of a 'continuous caring relationship' with an identified health care professional”[5] (p. 248). |

**Care Coordination** involved the characteristics noted in the following table.

| **Care Coordination**: Adapted from [6] |
| --- |
| Numerous participants are typically involved in care coordination; |
| Coordination is necessary when participants are dependent upon each other to carry out disparate activities in a patient's care; |
| In order to carry out these activities in a coordinated way, each participant needs adequate knowledge about their own and others' roles, and available resources; |
| In order to manage all required patient care activities, participants rely on (communication) exchange of information; |
| Integration of care activities has the goal of facilitating appropriate delivery of health care as well as social and community services. |

References:

1. Patient- and Family-Centered Care [<http://www.ipfcc.org/about/pfcc.html> ] Accessed….

2. Charles C, Gafni A, Whelan T: Shared decision-making in the medical encounter: What does it mean? (or it takes at least two to tango). Social Science & Medicine 1997, 44(5):681-692.

3. Levesque J-F, Harris MF, Russell G: Patient-centred access to health care: conceptualising access at the interface of health systems and populations. International Journal for Equity in Health 2013, 12(18).

4. Reid RH, J.McKendry, R. : Defusing the confusion: concepts and measures of continuity of healthcare. Final report. Prepared for the Canadian Health Services Research Foundation, the Canadian Institute for Health Information, and the Advisory Committee on Health Services of the Federal/Provincial/Territorial Deputy Ministers of Health. . In.; 2002.

5. Gulliford M, Naithani S, Morgan M: What is 'continuity of care'? Journal of Health Services Research & Policy 2006, 11(4):248-250.

6. McDonald KM, Sundaram V, Bravata DM, Lewis R, Lin N, Kraft SA, Owens DK: Closing the quality gap: A critical analysis of quality improvement strategies (Vol. 7: Care Coordination). In. Rockville, MD: Agency for Healthcare Research and Quality; 2007.
