# Supplementary material for: An evaluation study of caregiver perceptions of the Ontario’s Health Links program
Source: PLoS One. 2020 Feb 27;15(2):e0229579. doi: 10.1371/journal.pone.0229579 (PMC7046224; doi:10.1371/journal.pone.0229579)
Supplement: S2 Table — (DOCX) [file pone.0229579.s004.docx]

## **S2 Table**

## Caregiver’s Perceived Impacts of HL on Themselves and the HL Patients for Whom They Provide Care (n= 27)

| **Core Concept** | **Think about yourself as a caregiver. As a caregiver, HL has helped...** | **Mean (SD)* Scale 1-5** |
| --- | --- | --- |
| Care Coordination | Increase my knowledge of where to get health care services for myself. | 3.6 (1.56) |
| Care Coordination | Increase my knowledge of how to get health care services for myself. | 3.7 (1.49) |
| Overall Health Impact | Improve my physical health. | 2.8 (1.30) |
| Overall Health Impact | Improve my mental health. | 3.0 (1.21) |
| Overall Health Impact | Improve my quality of life. | 3.4 (1.25) |
| Patient and family-centred care | Increase my knowledge of how to best care for my loved one/friend. | 4.1 (1.09) |
| Patient and family centred care | Increase my involvement in the care decision-making for my loved one/friend. | 4.1 (1.27) |
| **Core Concept** | **Now, think about your loved one/friend to whom you provide care, HL has...** | **Mean (SD)* Scale 1-5** |
| Accessibility to care | Ensured there is minimal wait time for my loved one/friend to get services. | 4.2 (1.26) |
| Continuity of care | Ensured there is minimal disruption in the care provided to my loved one/friend. | 4.2 (1.17) |
| Continuity of care | Ensured the same people take care of my loved one/friend. | 4.1 (1.19) |
| Patient and family-centred care | Communicated well with my loved one/friend. | 4.2 (1.19) |
| Patient and family-centred care | Helped my loved one/friend better manage his/her own care. | 3.7 (1.18) |
| Patient and family-centred care | Provided services that meets my loved one’s/friend’s needs. | 4.2 (1.26) |
| Accessibility to Care | Provided services for my loved one/friend that are easy to access. | 4.1 (1.30) |
| Care Coordination | Provided services for my loved one/friend that are well coordinated. | 4.3 (1.16) |

*Responses on a scale from *1* *(strongly disagree)* to *5 (strongly*
